# Supplementary material for: Prehabilitation to prevent complications after cardiac surgery - A retrospective study with propensity score analysis
Source: PLoS One. 2021 Jul 16;16(7):e0253459. doi: 10.1371/journal.pone.0253459 (PMC8284810; doi:10.1371/journal.pone.0253459)
Supplement: S1 Appendix — (DOCX) [file pone.0253459.s001.docx]

**S1 Appendix - univariate analyses**

Table A – P-values of univariate analyses of the baseline characteristics in association with the outcomes (cardiac surgery complications),
n = 880

| characteristics | AF | Delirium | Lung infection | Prolonged MV | Re-admission ICU | Surgical re-exploration | Deep sternum wound | 30-day mortality |
| --- | --- | --- | --- | --- | --- | --- | --- | --- |
| Gender | 0.13 | *0.059* | 0.237 | 0.144 | 0.020* | 0.640 | 0.729 | 0.006* |
| Age | P<0.001* | P<0.001* | 0.422 | 0.949 | 0.776 | 0.665 | *0.055* | *0.069* |
| BMI | 0.299 | 0.785 | 0.131 | 0.729 | 0.489 | 0.555 | 0.244 | 0.966 |
| LVEF | 0.401 | 0.025* | 0.047 | 0.001* | 0.396 | 0.614 | 0.318 | 0.006* |
| NYHA | 0.629 | 0.020* | 0.007* | 0.004* | 0.026 | 0.180 | 0.723 | 0.433 |
| Logistic Euroscore II | 0.346 | P<0.001* | 0.190 | P<0.001* | 0.006* | P<0.001* | 0.387 | P<0.001* |
| Waiting time | 0.267 | 0.764 | 0.116 | 0.394 | 0.216 | 0.904 | 0.724 | 0.543 |
| Chronic lung disease | 0.028* | 0.319 | *0.086* | 0.810 | 0.003* | 0.375 | 0.002* | 0.010* |
| Diabetes mellitus | 0.919 | 0.493 | 0.636 | 0.677 | 0.898 | 0.227 | 0.305 | 0.618 |
| AF in history | 0.995 | *0.071* | 0.218 | 0.024* | *0.054* | 0.108 | 0.996 | 0.938 |
| Recent MI | 0.319 | 0.533 | 0.398 | 0.314 | 0.670 | 0.396 | 0.925 | 0.997 |
| Previous PCI | 0.976 | 0.626 | 0.210 | 0.214 | 0.579 | 0.917 | *0.055* | 0.946 |
| Previous cardiac surgery | *0.065** | 0.296 | 0.159 | 0.025* | 0.704 | 0.977 | 0.517 | 0.461 |
| History of CVA | 0.170 | 0.002* | 0.047* | 0.006* | 0.123 | 0.010* | 0.736 | 0.110 |
| Complexity of surgery | 0.323 | P<0.001* | 0.254 | P<0.001* | P<0.001* | P<0.001* | 0.622 | P<0.001* |
| Referred hospital | 0.506 | 0.808 | 0.416 | 0.304 | 0.483 | 0.884 | 0.318 | 0.320 |

Green: variables that were significantly associated with at least one of the postoperative complications and which were used to estimate the propensity score; * P-value < 0.05.
