# Supplementary material for: Prehabilitation to prevent complications after cardiac surgery - A retrospective study with propensity score analysis
Source: PLoS One. 2021 Jul 16;16(7):e0253459. doi: 10.1371/journal.pone.0253459 (PMC8284810; doi:10.1371/journal.pone.0253459)
Supplement: S2 Appendix — (DOCX) [file pone.0253459.s002.docx]

**S2 Appendix – balance between matched and unmatched data**Table A – Unstandardized values of balance before and after Nearest Neighbor matching, using propensity score, Greedy, no replacement, no caliper distance, ratio 1:3

matchit(formula = group ~ gender + age + lvef + euroscoreii_log + chr_lungdisease + AF in history + previous_cardiochir + previous_cvar + complexity_of_surgery + nyha, data = DataForMatch, method = "nearest", ratio = 3)

**Summary of balance for all data:**

Means Treated Means Control SD Control Mean Diff eQQ Med eQQ Mean eQQ Max

distance 0.1264 0.1008 0.0461 0.0257 0.0249 0.0259 0.0446

gender 0.7802 0.6958 0.4604 0.0844 0.0000 0.0879 1.0000

age 64.5275 66.0203 9.7663 -1.4928 2.0000 1.8022 5.0000

lvef 26.7033 27.1483 4.9725 -0.4450 0.0000 0.4396 10.0000

euroscoreii_log 2.0479 2.4426 2.8372 -0.3947 0.2320 0.5666 21.4580

chr_lung_disease 0.0769 0.1584 0.3654 -0.0815 0.0000 0.0879 1.0000

AF in history 0.1978 0.1901 0.3926 0.0077 0.0000 0.0000 0.0000

Previous_cardiochir 0.0330 0.0456 0.2088 -0.0127 0.0000 0.0110 1.0000

Previous_cva 0.0549 0.0608 0.2392 -0.0059 0.0000 0.0110 1.0000

Complexity of surgery 13.4725 13.5817 5.4300 -0.1092 0.0000 0.6484 10.0000

nyha 2.2637 2.5082 0.6503 -0.2445 0.0000 0.2418 1.0000

bmi 27.7140 27.6272 4.2951 0.0867 0.3350 0.4470 3.7590

waiting time 61.8571 59.8885 36.5549 1.9687 13.0000 15.8352 89.0000

diabetes 0.2198 0.2104 0.4078 0.0094 0.0000 0.0110 1.0000

recent myocardial infarction 0.0879 0.0748 0.2632 0.0131 0.0000 0.0110 1.0000

previous_pci 0.2527 0.1825 0.3865 0.0702 0.0000 0.0659 1.0000

**Summary of balance for matched data:**

Means Treated Means Control SD Control Mean Diff eQQ Med eQQ Mean eQQ Max

distance 0.1264 0.1262 0.0499 0.0002 0.0003 0.0006 0.0068

gender 0.7802 0.7546 0.4311 0.0256 0.0000 0.0330 1.0000

age 64.5275 64.7692 9.7980 -0.2418 1.0000 0.8132 5.0000

lvef 26.7033 26.7033 5.2974 0.0000 0.0000 0.1099 10.0000

euroscoreii_log 2.0479 2.1025 2.3807 -0.0546 0.0760 0.3352 16.5100

chr_lung_disease 0.0769 0.0842 0.2783 -0.0073 0.0000 0.0110 1.0000

AF in history 0.1978 0.2015 0.4018 -0.0037 0.0000 0.0000 0.0000

Previous_cardiochir 0.0330 0.0366 0.1882 -0.0037 0.0000 0.0000 0.0000

Previous_cva 0.0549 0.0549 0.2283 0.0000 0.0000 0.0000 0.0000

Complexity of surgery 13.4725 13.4432 5.5051 0.0293 0.0000 0.7912 10.0000

nyha 2.2637 2.2308 0.6655 0.0330 0.0000 0.0659 1.0000

bmi 27.7140 27.7258 4.3396 -0.0118 0.3530 0.5045 3.3830

waiting time 61.8571 59.0952 32.5543 2.7619 13.0000 14.9451 56.0000

diabetes 0.2198 0.2088 0.4072 0.0110 0.0000 0.0110 1.0000

recent myocardial infarction 0.0879 0.0696 0.2549 0.0183 0.0000 0.0220 1.0000

previous_pci 0.2527 0.1832 0.3875 0.0696 0.0000 0.0659 1.0000

**Percent Balance Improvement:**

Mean Diff. eQQ Med eQQ Mean eQQ Max

Sample sizes:

Control Treated

All 789 91

Matched 273 91

Unmatched 516 0

Discarded 0 0

distance 99.2451 98.8551 97.6339 84.7715

gender 69.6205 0.0000 62.5000 0.0000

age 83.8051 50.0000 54.8780 0.0000

lvef 100.0000 0.0000 75.0000 0.0000

euroscoreii_log 86.1550 67.2414 40.8375 23.0590

chr_lung_disease 91.0116 0.0000 87.5000 0.0000

AF in history 52.3551 0.0000 0.0000 0.0000

Previous_cardiochir 71.0671 0.0000 100.0000 100.0000

Previous_cva 100.0000 0.0000 100.0000 100.0000

Complexity of surgery 73.1701 0.0000 -22.0339 0.0000

nyha 86.5167 0.0000 72.7273 0.0000

bmi 86.3974 -5.3731 -12.8414 10.0027

waiting time -40.2925 0.0000 5.6211 37.0787

diabetes -17.0623 0.0000 0.0000 0.0000

recent myocardial infarct -39.4486 0.0000 -100.0000 0.0000

previous_pci 0.9122 0.0000 0.0000 0.0000

Table B – Standardized values of balance before and after Nearest Neighbor matching, using propensity score, Greedy, no replacement, no caliper distance, ratio 1:3

matchit(formula = group ~ gender + age + lvef + euroscoreii_log + chr_lung_disease + AF in history + Previous_cardiochir + Previous_cva + Complexity of surgery + nyha, data = DataForMatch, method = "nearest", ratio = 3)

**Summary of balance for all data:**

Means Treated Means Control SD Control Std. Mean Diff. eCDF Med eCDF Mean eCDF Max

distance 0.1264 0.1008 0.0461 0.5110 0.1608 0.1608 0.3020

gender 0.7802 0.6958 0.4604 0.2027 0.0422 0.0422 0.0844

age 64.5275 66.0203 9.7663 -0.1574 0.0178 0.0324 0.1182

lvef 26.7033 27.1483 4.9725 -0.0826 0.0114 0.0148 0.0331

euroscoreii_log 2.0479 2.4426 2.8372 -0.2051 0.0611 0.0602 0.1438

chr_lung_disease 0.0769 0.1584 0.3654 -0.3042 0.0408 0.0408 0.0815

AF in history 0.1978 0.1901 0.3926 0.0192 0.0038 0.0038 0.0077

Previous_cardiochir 0.0330 0.0456 0.2088 -0.0705 0.0063 0.0063 0.0127

Previous_cva 0.0549 0.0608 0.2392 -0.0257 0.0029 0.0029 0.0059

Complexity of surgery 13.4725 13.5817 5.4300 -0.0215 0.0269 0.0434 0.1198

nyha 2.2637 2.5082 0.6503 -0.3884 0.0076 0.0611 0.2293

bmi 27.7140 27.6272 4.2951 0.0189 0.0203 0.0213 0.0594

waiting time 61.8571 59.8885 36.5549 0.0741 0.0841 0.0970 0.3346

diabetes 0.2198 0.2104 0.4078 0.0225 0.0047 0.0047 0.0094

recent myocardial infarction 0.0879 0.0748 0.2632 0.0461 0.0066 0.0066 0.0131

previous_pci 0.2527 0.1825 0.3865 0.1607 0.0351 0.0351 0.0702

**Summary of balance for matched data:**

Means Treated Means Control SD Control Std. Mean Diff. eCDF Med eCDF Mean eCDF Max

distance 0.1264 0.1262 0.0499 0.0039 0.0037 0.0061 0.0403

gender 0.7802 0.7546 0.4311 0.0616 0.0128 0.0128 0.0256

age 64.5275 64.7692 9.7980 -0.0255 0.0147 0.0167 0.0549

lvef 26.7033 26.7033 5.2974 0.0000 0.0037 0.0024 0.0037

euroscoreii_log 2.0479 2.1025 2.3807 -0.0284 0.0220 0.0247 0.0659

chr_lung_disease 0.0769 0.0842 0.2783 -0.0273 0.0037 0.0037 0.0073

AF in history 0.1978 0.2015 0.4018 -0.0091 0.0018 0.0018 0.0037

Previous_cardiochir 0.0330 0.0366 0.1882 -0.0204 0.0018 0.0018 0.0037

Previous_cva 0.0549 0.0549 0.2283 0.0000 0.0000 0.0000 0.0000

Complexity of surgery 13.4725 13.4432 5.5051 0.0058 0.0366 0.0366 0.0733

nyha 2.2637 2.2308 0.6655 0.0524 0.0110 0.0156 0.0403

bmi 27.7140 27.7258 4.3396 -0.0026 0.0220 0.0267 0.0806

waiting time 61.8571 59.0952 32.5543 0.1040 0.1099 0.1180 0.3297

diabetes 0.2198 0.2088 0.4072 0.0264 0.0055 0.0055 0.0110

recent myocardial infarction 0.0879 0.0696 0.2549 0.0643 0.0092 0.0092 0.0183

previous_pci 0.2527 0.1832 0.3875 0.1593 0.0348 0.0348 0.0696

**Percent Balance Improvement:**

Std. Mean Diff. eCDF Med eCDF Mean eCDF Max

distance 99.2451 97.7220 96.2204 86.6565

gender 69.6205 69.6205 69.6205 69.6205

age 83.8051 17.4902 48.6260 53.5062

Sample sizes:

Control Treated

All 789 91

Matched 273 91

Unmatched 516 0

Discarded 0 0

lvef 100.0000 67.9268 83.5368 88.9263

euroscoreii_log 86.1550 64.0465 58.9665 54.1546

chr_lung_disease 91.0116 91.0116 91.0116 91.0116

AF in history 52.3551 52.3551 52.3551 52.3551

Previous_cardiochir 71.0671 71.0671 71.0671 71.0671

Previous_cva 100.0000 100.0000 100.0000 100.0000

Complexity of surgery 73.1701 -36.2341 15.5766 38.8372

nyha 86.5167 -44.3733 74.5315 82.4262

bmi 86.3974 -8.5282 -25.2300 -35.6942

waiting time -40.2925 -30.7374 -21.6504 1.4694

diabetes -17.0623 -17.0623 -17.0623 -17.0623

recent myocardial infarction -39.4486 -39.4486 -39.4486 -39.4486

previous_pci 0.9122 0.9122 0.9122 0.9122
