# Supplementary material for: Prehabilitation to prevent complications after cardiac surgery - A retrospective study with propensity score analysis
Source: PLoS One. 2021 Jul 16;16(7):e0253459. doi: 10.1371/journal.pone.0253459 (PMC8284810; doi:10.1371/journal.pone.0253459)
Supplement: S3 Appendix — (DOCX) [file pone.0253459.s003.docx]

**S3 Appendix – Re-analysis of data from 2015 and onwards**

Table A: Preoperative patient baseline characteristics and characteristics at hospitalization for the PRE and unmatched SCI groups for the inclusion period 2015-2017

| Preoperative characteristics | Unmatched groups 2015-2017 | | |  |
| --- | --- | --- | --- | --- |
|  | **PRE group**  **(n=91)** | **SC group**  **(n=474)** | **P-value** |  |
| Gender n (% men)^a,b^ | 71 (78.0%) | 342 (72.2%) | 0.25 |  |
| Age (% ≥ 65 years)^a,b^ | 49 (53.8%) | 291 (61.4%) | 0.18 |  |
| BMI (mean ± SD)^c^ | 27.7 (4.6) | 27.7 (4.3) | 0.97 |  |
| Left ventricular function^a,d^ |  |  | 0.37 |  |
| Poor LVEF (<31%) | 3 (3.3%) | 8 (1.7%) |  |  |
| Moderate LVEF (31-50%) | 24 (26.4%) | 110 (23.2%) |  |  |
| Good LVEF (>50%) | 64 (70.3%) | 356 (75.1%) |  |  |
| NYHA class^a,d^  Class I  Class II  Class III  Class IV | 6 (6.6%)  58 (63.7%)  24 (26.4%)  3 (3.3%) | 20 (4.2%) 242 (51.1%)  198 (41.8%) 14 (3.0%) | 0.03* |  |
| Logistic Euroscore II^a,e^ | 1.3 (0.9, 2.7) | 1.5 (0.9, 2.7) | 0.18 |  |
| Waiting time (days)^e,f^ | 55.0 (51.0, 66.0) | 55.5 (36.0, 82.0) | 0.27 |  |
| Chronic lung disease^a,b^ | 7 (7.7%) | 79 (16.7%) | 0.03* |  |
| Diabetes mellitus^b^ | 20 (22.0%) | 100 (21.1%) | 0.85 |  |
| Atrial fibirillation/flutter^a,b^ | 18 (19.8%) | 94 (19.8%) | 0.99 |  |
| Recent myocardial infarct^b^ | 8 (8.8%) | 25 (5.3%) | 0.19 |  |
| Previous PCI^b^ | 23 (25.3%) | 81 (17.1%) | 0.07 |  |
| Previous cardiac surgery^a,b^ | 3 (3.3%) | 23 (4.9%) | 0.52 |  |
| History of CVA^a,b^ | 5 (5.5%) | 37 (7.8%) | 0.44 |  |
| Characteristics at time of hospitalization | | | |  |
| Weight of surgery^a,d^  CABG isolated  Single, non CABG  whereof valve  whereof aortic  Two interventions  Three interventions | 47 (51.6%)  16 (17.6%)  15 (93.8)%  1 (6.3%)  26 (28.6%)  2 (2.2%) | 182 (38.4%) 165 (34.8%)  127 (95.0%)  9 (5.0%)  103 (21.7%) 24 (5.1%) | 0.003* |  |
| On-pump surgery^b^ | 52 (57.1%) | 341 (71.9%) | 0.005* |  |
| Bypass time (min)^e^ | 149.0 (112.0, 180.5) | 136.0 (105.0, 178.0) | 0.31 |  |
| Cross clamp time (min)^e^ | 97.0 (76.0, 132.0) | 91.0 (66.0, 126.0) | 0.33 |  |
| Surgery time (min)^e^ | 219.0 (182.0, 269.0) | 222.5 (184.0, 279.0) | 0.69 |  |
| Hospital Stay (days)^e^ | 6.0 (6.0, 10.0) | 7.0 (5.0, 8.0) | 0.45 | |
| Values are shown as median (Interquartile range) or n (% yes), unless otherwise noted. ^a^ Variable significantly associated with the outcomes and included in the logistic regression model for the propensity score; ^b^ Pearson’s chi-squared test; ^c^ Two sample t test; ^d^ Fisher’s exact test; ^e^ Wilcoxon rank-sum test; ^f^ At time of acceptance for surgery till the day of surgery. BMI: Body mass index; CABG: Coronary artery bypass graft; CVA: Cerebral vascular accident; IQR: Interquartile range; LVEF: Left ventricular ejection fraction; NYHA: New York Heart Association; PCI: Percutaneous coronary intervention; PRE group: Prehabilitation group; SC group: Standard care group SD: Standard deviation. | | | | |

Table B: Incidence of in-hospital acquired postoperative complications

|  | Unmatched groups 2015-2017 | | |  |
| --- | --- | --- | --- | --- |
| Outcomes | **PRE group**  **(n=91)** | **SC group**  **(n=474)** | **P-value^a^** |  |
| Atrial  fibrillation/flutter | 13 (14.3%) | 111 (23.4%) | 0.05 |  |
| Delirium | 14 (15.4%) | 67 (14.1%) | 0.76 |  |
| Lung infection | 6 (6.6%) | 23 (4.9%) | 0.49 |  |
| Prolonged MV | 8 (8.8%) | 21 (4.4%) | 0.08 |  |
| Re-admission ICU | 9 (9.9%) | 27 (5.7%) | 0.13 |  |
| Surgical re-  exploration | 7 (7.7%) | 20 (4.2%) | 0.15 |  |
| Deep sternum  wound infection | 1 (1.1%) | 7 (1.5%) | 0.78 |  |
| 30-day mortality | 2 (2.2%) | 6 (1.3%) | 0.49 |  |

Values are shown as n (% yes). **^a^**Pearson’s chi-squared test; ICU: Intensive care unit; MV: Mechanical ventilation.
