# Supplementary material for: Prehabilitation to prevent complications after cardiac surgery - A retrospective study with propensity score analysis
Source: PLoS One. 2021 Jul 16;16(7):e0253459. doi: 10.1371/journal.pone.0253459 (PMC8284810; doi:10.1371/journal.pone.0253459)
Supplement: Attachment — Submitted filename: 2021-02-04 Reviewers response_PLOSONE_s2.docx [file pone.0253459.s004.docx]

University Medical Center Groningen

Hanzeplein 1, AB41,

9713 GZ Groningen

The Netherlands

Phone:+3150 3617527

Email: [j.hartog@umcg.nl](mailto:j.hartog@umcg.nl)

Robert Jeenchen Chen, MD, MPH	16 March 2021

Academic Editor
PLOS ONE
Email: [plosone@plos.org](mailto:plosone@plos.org).

Dear Professor Robert Jeenchen Chen:

On behalf of the authors, I thank you and the reviewers for the time and effort for examining our manuscript. Furthermore, we are thankful for the opportunity to submit a revised version of our manuscript “*Prehabilitation to prevent complications after cardiac surgery - A retrospective study with propensity score analysis*”. Since data and knowledge about the effect of prehabilitation in cardiac surgical patients is scarce, we think this retrospective analysis is a first and valuable indication of the safety and possible potential of prehabilitation in open heart surgery patients and valuable to share with the readership of PLOS ONE.

Based on your and the reviewers’ comments, the manuscript is adapted and improved. We used the track changes mode in MS Word to highlight the changes made in the manuscript. Please find attached our detailed response to the reviewers’ comments. Page numbers in this response refer to the version of the manuscript with track changes.

All authors have contributed significantly and have read and approved the submission of this revised manuscript. We hope our revised manuscript is suitable for publication in PLOS ONE.

On behalf of all authors,

Yours sincerely,

Johanneke Hartog, MSc

*PhD-candidate Cardiology and Thoracic surgery*

PONE-D-20-40737 - Prehabilitation to prevent complications after cardiac surgery - A retrospective study PLOS ONE

## Journal Requirements:

When submitting your revision, we need you to address these additional requirements.

1. Please ensure that your manuscript meets PLOS ONE's style requirements, including those for file naming. The PLOS ONE style templates can be found at

https://journals.plos.org/plosone/s/file?id=wjVg/PLOSOne_formatting_sample_main_body.pdf and

https://journals.plos.org/plosone/s/file?id=ba62/PLOSOne_formatting_sample_title_authors_affiliations.pdf

**Reply:** Thank you for the observation. We changed the following aspects:

- The title page has been adapted to the PLOS ONE’s style requirements.

- The font style has been changed to ‘Italics’

- Level 1 and 2 headings are used for the headers throughout the manuscript in respectively ‘Italics, Bold typ, 18pt font’ and ‘Italics, Bold typ, 16pt font’.

2. In the ethics statement in the manuscript and in the online submission form, please provide additional information about the patient records/samples used in your retrospective study, including the date range (month and year) during which patients' medical records/samples were accessed.

**Reply:** We added in the Materials and methods section ‘*Data were collected between March 2016 and February 2018 from existing databases (the Dutch registration database and the database of the intensive care unit) and medical records.*’ (p.5, line 88-89). We removed the sentence ‘*Data were collected from existing databases and medical records.*’ from the sub header ‘outcomes’ in the Materials and methods section (p.6, line 121, 122)

3. Please include captions for your Supporting Information files at the end of your manuscript, and update any in-text citations to match accordingly. Please see our Supporting Information guidelines for more information: http://journals.plos.org/plosone/s/supporting-information.

**Reply:** Thank you for your notice:
- The section ‘Supporting Information’ has been added to the end of the manuscript, including the S1-S3 Appendix. The filenames of the supporting information files are changed into ‘S1 Appendix - Univariate analyses’, ‘S2 Appendix – Balance between matched and unmatched data’, and ‘S3 Appendix – Re-analysis of data from 2015 and onwards’ (p.17, line 336-339).
- The citations in the text are updated as well.

4.We note that the grant information you provided in the ‘Funding Information’ and ‘Financial Disclosure’ sections do not match.

**Reply:** We have adjusted the ‘Funding information’ so that it matched with ‘Financial Disclosure’ now. In addition, we add the sentence ‘*The funders had no role in study design, data collection and analysis, decision to publish, or preparation of the manuscript*.’ to the Financial Closure Statement section in the manuscript (p.17, line 328-330).

When you resubmit, please ensure that you provide the correct grant numbers for the awards you received for your study in the ‘Funding Information’ section.

**Reply:** We provided the correct grant numbers for Edwards Lifesciences and ‘Stichting Beatrixoord Noord-Nederland’. The grant number for SA, Abbott (former St. Jude Medical Nederland B.V.) is not applicable.

5.Thank you for stating the following in the Competing Interests section:

"JH, SD, and MAM report grants from Edwards Lifesciences, SA, Abbott (former St. Jude Medical Nederland B.V.), and ‘Stichting Beatrixoord Noord-Nederland’. MAM reports grants from AtriCure, Getinge and consultancy from LivaNova. The remaining authors have nothing to disclose."

Please confirm that this does not alter your adherence to all PLOS ONE policies on sharing data and materials, by including the following statement: "This does not alter our adherence to PLOS ONE policies on sharing data and materials.” (as detailed online in our guide for authors http://journals.plos.org/plosone/s/competing-interests). If there are restrictions on sharing of data and/or materials, please state these. Please note that we cannot proceed with consideration of your article until this information has been declared.

Please include your updated Competing Interests statement in your cover letter; we will change the online submission form on your behalf.

**Reply:** The sentence ‘*This does not alter our adherence to PLOS ONE policies on sharing data and materials*’ is added to the Competing Interests section (p.17, line 331-335).

Please know it is PLOS ONE policy for corresponding authors to declare, on behalf of all authors, all potential competing interests for the purposes of transparency. PLOS defines a competing interest as anything that interferes with, or could reasonably be perceived as interfering with, the full and objective presentation, peer review, editorial decision-making, or publication of research or non-research articles submitted to one of the journals. Competing interests can be financial or non-financial, professional, or personal. Competing interests can arise in relationship to an organization or another person. Please follow this link to our website for more details on competing interests: http://journals.plos.org/plosone/s/competing-interests

## Reviewer's Responses to Questions

Comments to the Author

1. Is the manuscript technically sound, and do the data support the conclusions?

The manuscript must describe a technically sound piece of scientific research with data that supports the conclusions. Experiments must have been conducted rigorously, with appropriate controls, replication, and sample sizes. The conclusions must be drawn appropriately based on the data presented.

Reviewer #1: Yes

Reviewer #2: No

________________________________________

2. Has the statistical analysis been performed appropriately and rigorously?

Reviewer #1: Yes

Reviewer #2: Yes

________________________________________

3. Have the authors made all data underlying the findings in their manuscript fully available?

The PLOS Data policy requires authors to make all data underlying the findings described in their manuscript fully available without restriction, with rare exception (please refer to the Data Availability Statement in the manuscript PDF file). The data should be provided as part of the manuscript or its supporting information, or deposited to a public repository. For example, in addition to summary statistics, the data points behind means, medians and variance measures should be available. If there are restrictions on publicly sharing data—e.g. participant privacy or use of data from a third party—those must be specified.

Reviewer #1: Yes

Reviewer #2: Yes

________________________________________

4. Is the manuscript presented in an intelligible fashion and written in standard English?

PLOS ONE does not copyedit accepted manuscripts, so the language in submitted articles must be clear, correct, and unambiguous. Any typographical or grammatical errors should be corrected at revision, so please note any specific errors here.

Reviewer #1: Yes

Reviewer #2: No

**Reply:** Thank you for this observation. The manuscript has been reviewed on textual grammar (textual changes are highlighted in yellow).

________________________________________

5. Review Comments to the Author

Please use the space provided to explain your answers to the questions above. You may also include additional comments for the author, including concerns about dual publication, research ethics, or publication ethics. (Please upload your review as an attachment if it exceeds 20,000 characters)

Reviewer #1: Dear the authors of the manuscript entitled "Prehabilitation to prevent complications after cardiac surgery - A retrospective study"

Thank you for doing this study

I think this study is an excellent one since it highlights the importance of preoperative rehabilitation program in decreasing the incidence of postoperative complications after elective cardiac surgery

This study demonstrated that there was clear benefit of exercise programs in in decreasing the incidence of atrial fibrillation after cardiac surgery, however it did not show a clear benefit in improving the other outcomes

there are some limitations of this study and there were mentioned by the authors, however I think this study had good power since it included all comers and had propensity matched groups

All in all I have no concerns about this manuscript

Thank you

**Reply:** Thank you for taking the time to review our manuscript and for the positive words about this study. We agree with you about the importance of preoperative rehabilitation. We hope that the results of this study will help to increase the awareness and understanding of the effects of prehabilitation and that it will encouraged clinicians and scientists to take the possibility of prehabilitation into account. Especially, now the risk factors, such as age, physical inactivity, obesity, diabetes, hypertension, and dyslipidaemia, are steadily increasing in patients undergoing cardiac surgery[1,2].

Reviewer #2: This study is a single-center, retrospective study that aims to explore the impact of a prehabilitation program on short-term outcomes following cardiac surgery. This is an interesting study of ultimately 364 patients spanning between 2014 to 2017. The authors found that patients that underwent prehabilitation had a lower incidence of atrial fibrillation (14.3% vs 25.3%) compared to the standard of care. Comments and questions are enumerated.

**Reply:** Thank you for your interest in this study and for your effort and time examining the manuscript. Please find below the answers of your comments and questions.

1. A major limitation of this study is the lack of randomization of the two treatment arms. This was addressed in the discussion section but may benefit from earlier introduction in the methods section. Propensity matching was utilized to account for patient and hospital differences but unfortunately this does not account for bias such as assessment of patient functional status.

**Reply (response to the first part of the question):** We agree that randomized controlled trials are preferred over observational studies to evaluate a new intervention, but observational designs have also their strengths. For example, an observational study includes an all-comers population in a natural setting. In addition, it’s explorative nature, though less controlled and powerful, helps to develop theory and potential future hypotheses. We used this study as an explorative study for eventually setting up such a randomized controlled trial to further systematically investigate the effects of prehabilitation on functional status, postoperative surgical complications, major adverse cardiac events, cardiorespiratory fitness, muscle strength, and mental health in a randomized and controlled manner[3]. Since data and knowledge about the effect of prehabilitation in cardiac surgical patients is scarce, we think this retrospective analysis is a first and valuable indication of the safety and possible potential of prehabilitation in open heart surgery patients and valuable to share with the readership of PLOS ONE.

Changes in manuscript:

Thank you for your suggestion, we added ‘with propensity score analysis’ in the title. In this way, the reader is informed early about the design of this study. In our opinion, limitations should be addressed in the Discussion section, therefore we did not remove this limitation from the Discussion section.

**Reply (response to second part of the question):**

Unfortunately, in this retrospective, exploratory study only outcomes that were collected in the standard care were available. However, patients who are physically constrained by their underlying cardiac condition are often operated with higher priority. By focussing on elective patients, all patients were able to carry out activities of daily living, independently .

Although we do agree that there might be confounding factors that could have contributed to our findings, we think that we minimized these effects by using propensity score matching procedure. As advised in the literature[4]; we matched more patients from the SC group to each patient from the PRE group. Due to the considerably larger control group it was possible to find three comparable patients from the SC group for each individual patient in the PRE group. As a result, all baseline variables that potentially influenced the outcomes were well balanced between the matched SC group and the PRE group after this propensity score matching. As such, we think we chose an appropriate method to minimize the potential bias in this retrospective observational study, but, as we stated in the Discussion section, the current results should be confirmed by randomized controlled trials.

2. Did the authors run a power analysis? The study’s sample size was very small for both treatment arms after propensity matching. The outcomes of interest such as mortality, sternum wound infection, lung infection and surgical re-exploration were rare events and may have not occurred enough to run a robust analysis. Characteristics such as patients who underwent aortic surgery were also rare events.

**Reply:** Thank you for your question. Because of the retrospective nature, we did not perform a power analysis. Indeed results of this study should be interpreted with caution, therefore we stated in the Discussion section that these results should be confirmed by well-powered randomized controlled studies (p.13, line 241). This study was however done in preparation to set up an actual prospective randomized controlled trial[3], with among others the current data we performed a power analysis for this prospective randomized controlled trial. The group who underwent aortic surgery is not well represented in the study, because it is a less common operation.

Changes to manuscript:

- ‘of the study’ has been added to the third paragraph in the Discussion section (p.12, line 213).
- ‘Randomized controlled trials’ has been added to the last paragraph in the Discussion section (p.13, line 241).

3. Were the authors able to run a sub-analysis of the patients that experienced postoperative atrial fibrillation? While the authors proposed that inflammation may be a major driver of the difference in this complication, it would be interesting to see how functional status, comorbidities, complexity of surgery, cross clamp and bypass times compared in these patients.

**Reply:** We thank the reviewer for the interesting question. If we interpret this question correctly, the reviewer suggests to investigate whether there are other factors besides inflammation that may influence the occurrence of atrial fibrillation. A comparison between the patients with postoperative atrial fibrillation (POAF) of the PRE-group and the patients with POAF of the SC-group for the variables, functional status, comorbidities, complexity of surgery, cross clamp and bypass times, is suggested as sub-analysis.

Indeed, as stated in the Discussion section (p.11, line 201,202) the mechanisms resulting in POAF are multifactorial[5]. Univariate analyses, which we conducted in preparation of the propensity analysis, showed that factors such as chronic lung disease and previous cardiac surgery were (borderline) predictors of POAF, while NYHA class and the complexity of the surgery were no predictors of POAF (S1 Appendix – univariate analyses). Furthermore, we agree with the reviewer that the use and duration of cardiopulmonary bypass can up-regulate the body’s inflammatory response to heart surgery resulting in a higher incidence of POAF. Since a sub-analysis on patients with POAF would downgrade the study power (since the sample size for the PRE-group only will be 13 patients), we have chosen to perform a propensity-score matching analysis. After matching the standardized differences, all above mentioned variables were less than 0.1 indicating a negligible difference between the PRE-group and matched SC-group. Despite the good balance in these variables between the groups, there was still a significant difference on POAF, assuming that prehabilitation is also contributing in reducing the incidence of POAF.

It is well stated that, besides postoperative inflammatory markers, preoperative inflammatory markers are also risk factors in the development of POAF, regardless the use of a cardiopulmonary bypass[5-8]. On the other hand, the anti-inflammatory effects of exercise, healthy food, and stress reduction are well known[9-11]. Therefore, we find it interesting to research whether there might be a causal relation between the possible anti-inflammatory effects of the Heart-ROCQ-pilot program on the pre- and postoperative inflammatory state and the risks of AF.

Changes in manuscript:

- The sentences ‘*The development of postoperative AF has been associated with an increase of pre- and postoperative inflammatory markers[1]. It is likely that inflammation is an important factor in the multifactorial pathophysiological mechanisms that cause postoperative AF. It is possible that exercise, healthy food, and psychological stress reduction (offered to the PRE-group) down-regulated the preoperative inflammatory state, whereby the inflammatory response from cardiac surgery was modulated, reducing the risk of AF[23–26]. However, these possible mechanisms should be investigated further.*’ are changed into ‘*The development of postoperative AF has, regardless the use of a cardiopulmonary bypass, been associated with an increase of preoperative and postoperative inflammatory markers[1, 23]. Inflammation is therefore suggested as an important factor in the multifactorial pathophysiological mechanisms that cause postoperative AF[24]. On the other hand, the anti-inflammatory effects of exercise, healthy food, and psychological stress reduction (offered to the PRE-group) are well stated[25–27]. Therefore, it would be interesting to research whether there might be a causal relation between the possible anti-inflammatory effects of the Heart-ROCQ-pilot program on the pre- and postoperative inflammatory state and the risks of AF.*’ in the second paragraph of the Discussion section (p.12,13, line 199-208)

4. Minor: The Diagnostic and Statistical Manual of Mental Disorders 5th edition is the current standard.

**Reply:** We agree with the reviewer. In the University Medical Center Groningen, delirium is currently diagnosed according to the criteria of the Diagnostic and Statistical Manual of Mental Disorders 5^th^ edition.

________________________________________

6. PLOS authors have the option to publish the peer review history of their article (what does this mean?). If published, this will include your full peer review and any attached files.

If you choose “no”, your identity will remain anonymous but your review may still be made public.

Do you want your identity to be public for this peer review? For information about this choice, including consent withdrawal, please see our Privacy Policy.

Reviewer #1: Yes: Salah Eldien Altarabsheh

Reviewer #2: No

## References

1	Ghanta RK, Kaneko T, Gammie JS, et al. Evolving trends of reoperative coronary artery bypass grafting: An analysis of the Society of Thoracic Surgeons Adult Cardiac Surgery Database. J Thorac Cardiovasc Surg 2013;145:364–372.

2	Kindo M, Hoang Minh T, Perrier S, et al. Trends in isolated coronary artery bypass grafting over the last decade. Interact Cardiovasc Thorac Surg 2017;24:71–76.

3	Hartog J, Blokzijl F, Dijkstra S, et al. Heart Rehabilitation in patients awaiting Open heart surgery targeting to prevent Complications and to improve Quality of life (Heart-ROCQ): study protocol for a prospective, randomised, open, blinded endpoint (PROBE) trial 2019;9:e031738.

4	Deb S, Austin PC, Tu J V., et al. A Review of Propensity-Score Methods and Their Use in Cardiovascular Research. Can J Cardiol 2016;32:259–265.

5	Boons J, Van Biesen S, Fivez T, et al. Mechanisms, Prevention, and Treatment of Atrial Fibrillation After Cardiac Surgery: A Narrative Review. J Cardiothorac Vasc Anesth 2020;000:1–10.

6	Lo B, Fijnheer R, Nierich AP, et al. C-reactive protein is a risk indicator for atrial fibrillation after myocardial revascularization. Ann Thorac Surg 2005;79:1530–1535.

7	Weymann A, Popov AF, Sabashnikov A, et al. Baseline and postoperative levels of C-reactive protein and interleukins as inflammatory predictors of atrial fibrillation following cardiac surgery: a systematic review and meta-analysis. Kardiol Pol 2018;76:440–451.

8	Todorov H, Janssen I, Honndorf S, et al. Clinical significance and risk factors for new onset and recurring atrial fibrillation following cardiac surgery - a retrospective data analysis. BMC Anesthesiol 2017;17:1–10.

9	Giugliano D, Ceriello A, Esposito K. The Effects of Diet on Inflammation. Emphasis on the Metabolic Syndrome. J Am Coll Cardiol 2006;48:677–685.

10	Zanchi NE, Almeida FN, Lira FS, et al. Renewed avenues through exercise muscle contractility and inflammatory status. ScientificWorldJournal 2012;2012:1–7.

11	Wirtz PH, von Känel R. Psychological Stress, Inflammation, and Coronary Heart Disease. Curr Cardiol Rep 2017;19:1–10.
