# Supplementary material for: Prehabilitation to prevent complications after cardiac surgery - A retrospective study with propensity score analysis
Source: PLoS One. 2021 Jul 16;16(7):e0253459. doi: 10.1371/journal.pone.0253459 (PMC8284810; doi:10.1371/journal.pone.0253459)
Supplement: Attachment — Submitted filename: 2021-05-25_review respons_PLOSONE_s3_PONE-D-20-40737R1.docx [file pone.0253459.s005.docx]

University Medical Center Groningen

Hanzeplein 1, AB41,

9713 GZ Groningen

The Netherlands

Phone:+3150 3617527

Email: [j.hartog@umcg.nl](mailto:j.hartog@umcg.nl)

Robert Jeenchen Chen, MD, MPH	25 May 2021

Academic Editor
PLOS ONE
Email: [plosone@plos.org](mailto:plosone@plos.org).

Dear Professor Robert Jeenchen Chen:

Thank you for the opportunity to submit a second revised version of our manuscript “*Prehabilitation to prevent complications after cardiac surgery - A retrospective study with propensity score analysis*”.

Based on the reviewers’ comments, the manuscript has been adapted. We used the track changes mode in MS Word to highlight the changes made in the manuscript. Please find attached our detailed response to the reviewers’ comments. Page numbers in this response refer to the version of the manuscript with track changes.

We think that our manuscript has improved considerably. All authors have contributed significantly and have read and approved the submission of this revised manuscript. We hope our revised manuscript is suitable for publication in PLOS ONE.

On behalf of all authors,

Yours sincerely,

Johanneke Hartog, MSc

*PhD-candidate Cardiology and Thoracic surgery*

PONE-D-20-40737R1 - Prehabilitation to prevent complications after cardiac surgery - A retrospective study with propensity score analysis

## Reviewers' comments:

Reviewer's Responses to Questions

Comments to the Author

1. If the authors have adequately addressed your comments raised in a previous round of review and you feel that this manuscript is now acceptable for publication, you may indicate that here to bypass the “Comments to the Author” section, enter your conflict of interest statement in the “Confidential to Editor” section, and submit your "Accept" recommendation.

Reviewer #1: All comments have been addressed

Reviewer #3: All comments have been addressed

________________________________________

2. Is the manuscript technically sound, and do the data support the conclusions?

The manuscript must describe a technically sound piece of scientific research with data that supports the conclusions. Experiments must have been conducted rigorously, with appropriate controls, replication, and sample sizes. The conclusions must be drawn appropriately based on the data presented.

Reviewer #1: Yes

Reviewer #3: Partly

________________________________________

3. Has the statistical analysis been performed appropriately and rigorously?

Reviewer #1: Yes

Reviewer #3: Yes

________________________________________

4. Have the authors made all data underlying the findings in their manuscript fully available?

The PLOS Data policy requires authors to make all data underlying the findings described in their manuscript fully available without restriction, with rare exception (please refer to the Data Availability Statement in the manuscript PDF file). The data should be provided as part of the manuscript or its supporting information, or deposited to a public repository. For example, in addition to summary statistics, the data points behind means, medians and variance measures should be available. If there are restrictions on publicly sharing data—e.g. participant privacy or use of data from a third party—those must be specified.

Reviewer #1: Yes

Reviewer #3: Yes

________________________________________

5. Is the manuscript presented in an intelligible fashion and written in standard English?

PLOS ONE does not copyedit accepted manuscripts, so the language in submitted articles must be clear, correct, and unambiguous. Any typographical or grammatical errors should be corrected at revision, so please note any specific errors here.

Reviewer #1: Yes

Reviewer #3: Yes

________________________________________

6. Review Comments to the Author

Please use the space provided to explain your answers to the questions above. You may also include additional comments for the author, including concerns about dual publication, research ethics, or publication ethics. (Please upload your review as an attachment if it exceeds 20,000 characters)

Reviewer #1: Dear the authors

Thank you again for taking all considerations for the reviewers comments

I have no concerns about this manuscript

**Reply:** Thank you for your effort to review our manuscript.

Reviewer #3: Introduction:

- The introduction does not cover all the elements of the study.

- I suggest the authors expose to the following points in the introduction: What is known about the cardiac surgery? What is not known? Why the study was done?".

**Reply:** Thank you for this suggestion. We reviewed the introduction thoughtfully, please find our comments and revisions regarding your suggestions below.

What is known about the cardiac surgery

In the first and second paragraph we focus mainly on what is known about cardiac surgery with respect to what is relevant to our research question. It describes the impact of the surgery nowadays (first paragraph) and how the risk on postoperative complications is linked to the patients’ preoperative status (second paragraph).

Changes to manuscript

- To give more background information about cardiac surgery we added ‘*More than 15,000 patients with ischemic heart disease, which is the leading cause of death in Western countries, undergo a coronary artery bypass graft (CABG), valve, and/or aortic surgery in the Netherlands[1,2].*’ (Paragraph 1, p.3, line: 46-47) and the second sentence was changed from ‘Cardiac surgery is a major intervention, and despite the recent improvements in operative care, the risks of postoperative complications are still high.’ into ‘*Although mortality has decreased due to improvements in operative care, the risks of postoperative complications are still high.*’ (paragraph 1, p.3, line: 47-48).
- In addition, ‘*it is well known that*’ is added to the first sentence of the second paragraph to emphasize that this paragraph is about what is already known (p.3, line: 55).

What is not known

In the third paragraph we indicated the current knowledge gap among the effects of prehabilitation in cardiac surgical patients. It is stated that the literature mainly consists of small trials, which are mainly focused on inspiratory muscle training as prehabilitation program. In addition, it is stated that the effects of a multidisciplinary prehabilitation program remain unclear.

Changes to manuscript

- To clarify this point we changed the sentences ‘However, the evidence for the benefits and risks of multidisciplinary --- (e.g. arrhythmias or delirium) remain unclear.’ into ‘*However, the evidence for the benefits and risks of preoperative CR (prehabilitation) is scarce. Evidence is usually derived from small trials investigating the effect of a single-component prehabilitation program, which often consist of inspiratory muscle training (IMT), on pulmonary complications, length of stay, and mortality[10,11]. A multidisciplinary prehabilitation program consisting of different components (e.g. whole body exercise, dietary’, and psychological guidance) can be effective on multiple aspects and might therefore be more effective than a single-component program. Since very little is known about the unintended consequences and effects of such multidisciplinary program, it is relevant to explore the effects of a multidisciplinary prehabilitation program on pulmonary complications as well as on other postoperative complications such as arrhythmias or delirium.*’ (Third paragraph, p.3-4, line: 63-75).

Why the study was done

We emphasized the importance of this study by mentioning the high number of postoperative complications and these consequences in the first paragraph (p.3, line: 49-52) and mention the association between preoperative status and the risk of postoperative complications (Paragraph 2, P.3, line: 55-62). To accentuate the aspect of why the study was done, we did the below mentioned revisions.

Changes to manuscript

- We added the sentence ‘*A decline in postoperative complications would thus reduce the patient burden and healthcare costs. Therefore, it is important to explore whether the risks of complications can be reduced before surgery.*’ (p.3, line: 53-54).
- We moved the reason why cardiac rehabilitation or prehabilitation is suggested forward into the third paragraph (p.3, line: 63-66). The first sentence of this paragraph is thus changed from ‘Cardiac rehabilitation (CR) aims “to influence favourably the underlying cause of cardiovascular disease, as well as to provide the best possible physical, mental and social conditions”[9] and therefore has been suggested to improve the preoperative status of the patient.’ into ‘*Cardiac rehabilitation (CR) has been suggested to improve the preoperative status of the patient and therefore be potentially effective in preventing postoperative complications. The aim of CR is “to influence favourably the underlying cause of cardiovascular disease, as well as to provide the best possible physical, mental and social conditions”[9].*’ (p.3, line: 63-66).
- The added sentence ‘*A prehabilitation program consisting of more components (e.g. whole body exercise, dietary, and psychological guidance) can be effective on multiple aspects and might therefore be more effective than a single-component program.*’ also contributes to the rationale for conducting the current study (p.3-4, line: 70-72).

- Also, add the hypothesis of the study.

**Reply:** Thank you for the suggestion, we added ‘*Although this is an exploratory study, based on the shown effects of preoperative IMT[12,13], we expect a lower rate of in-hospital complications and no unintended consequences in patients who followed multidisciplinary prehabilitation.*’ to the end of the Introduction (p.3, line: 78-80).

Methods:

- The methods cannot be followed in this frame.

- Kindly, re-frame the methods in accordance with components (SPICES) for methods:

a. Study design, setting, sample size

b. Participant

c. Intervention/issue of interest (exposure)

d. Comparison

e. Ethics and endpoint

f. Statistical analysis

**Reply:** Thank you for the suggestion. As suggested, we reframed the section ‘MATERIALS AND METHODS’ in accordance with the SPICES components for methods and we reordered several sentences. Please find a detailed description of the changes to the manuscript below. These changes are also shown in the manuscript with track changes.

Changes to manuscript:

1. We added the header ‘STUDY DESIGN, SETTING, AND SAMPLE SIZE’ (p.4, line: 82).
2. The sample size ‘880’ has been added to the first line of the section ‘MATERIALS AND METHODS’ (p.4, line: 83).
3. We removed *‘(≥18 years)’, ‘(i.e. no main stem stenosis, unstable angina pectoris or progressive symptoms)’,* and *‘(i.e. coronary artery bypass graft [CABG], valve surgery, aortic surgery or combined procedures)’* from the first sentence of the section ‘MATERIALS AND METHODS’ (p.4, line: 83-85). The last two removed parts ‘*(i.e. no main stem stenosis, unstable angina pectoris or progressive symptoms),’* and *‘(i.e. coronary artery bypass graft [CABG], valve surgery, aortic surgery or combined procedures)*’ were included to the first sentence of the new header ‘PARTICIPANT’ and *‘(≥18 years)’* is changed into adults, see next item (item 4).
4. The header ‘PARTICIPANTS’ and the sentence ‘*Adults admitted for --- procedures were included.*’ were added to the section ‘MATERIALS AND METHODS’ (p.4-5, line: 95-97).
5. The sentences *‘The Heart Center of the UMCG --- the standard hospital protocol of the UMCG.’* were moved from the old header ‘PROCEDURE’ (p.5, line: 109-113) to the new header ‘STUDY DESIGN, SETTING, AND SAMPLE SIZE’ (p.4, line: 86-91).
6. ‘*However, in February 2015 a multidisciplinary preoperative and postoperative rehabilitation program (the Heart-ROCQ pilot program) was implemented in the UMCG for internally referred patients.’* was moved to the header ‘STUDY DESIGN, SETTING, AND SAMPLE SIZE’ (p.4, line: 91-92).
7. The sentences *‘This resulted in a --- during the waiting time.’* were added at the end of the header ‘STUDY DESIGN, SETTING, AND SAMPLE SIZE’ (p.4, line: 92-94).
8. We changed the header ‘PROCEDURE’ into ‘INTERVENTION’ (p.5, line: 108) and we added the header ‘COMPARISON’ (p.5, line:122).
9. The last sentence of the header ‘INTERVENTION’: ‘Patients who followed this program were referred to as the PRE-group.’ was changed into ‘*Patients who followed this program in addition to the standard surgical care were referred to as the PRE-group.*’ and was shifted one sentence forwards from line 119,120 to line 117,118 (p.5).
10. The sentences ‘*Before February 2015, no prehabilitation was offered --- an outpatient CR was offered as standard care in the referral hospital[14].*’ were moved to the new header ‘COMPARISON’ (p.6, line: 123-124).
11. The words ‘*and thus followed only the standard surgical care,*’ we added to the last sentence of the new header ‘COMPARISON’ (p.6, line: 126).
12. We changed the header ‘OUTCOMES’ into ‘ETHICS AND ENDPOINT’ (p.6, line: 142).
13. The sentences ‘*All collected data were de-identified --- the database of the intensive care unit) and medical records.*’ were moved from ‘MATERIALS AND METHODS’ (p.5, line: 103-106) to ‘ETHICS AND ENDPOINT’ (p.7, line: 150-153).
14. We changed the header ‘DATA ANALYSES’ into ‘STATISTICAL ANALYSES’ (p.8, line: 159).

Discussion:

Kindly, reframe it as the following:

a. Main findings of the present study

b. Comparison with other studies

c. Implication, and explanation of findings

d. Strengths and limitations

e. Conclusion, recommendation and future direction.

**Reply:** Thank you for this suggestion. We carefully went through the Discussion to check the order of topics, with the intention to follow up on this reviewer’s suggested order. However, we came to the conclusion that the current form of the Discussion almost has the same order as suggested. Only within topics ‘b’ and ‘c’ (i.e. ‘comparison with other studies’ and ‘implication, and explanation of findings’) are atrial fibrillation first described (second paragraph, p.13,14, line: 236-245) and followed by the other postoperative complications (third paragraph, p.14, line: 246-260). In addition, we chose to describe the limited sample size as a limitation in the third paragraph (p.14, line: 247-250) and not in the fourth paragraph, to improve the ‘flow’ of the text.

The order of the current Discussion section is thus (see also the remarks in the revised manuscript with tracking changes):

Paragraph 1:

        a. Main findings of the present study (p.13, line: 231-235)

Paragraph 2:

        b. Comparison with other studies – Atrial Fibrillation (p.13, line: 236-238)

        c. Implication, and explanation of findings – Atrial Fibrillation (p.13, line: 237-238 & p.13,14, line: 239-245)

Paragraph 3:

        b. Comparison with other studies – other postoperative complications (p.14, line: 250-253)

        c. Implication, and explanation of findings – other postoperative complications (p.14, line: 253-260)

Paragraph 4:

        d. Strengths and limitations (p.14-15, line: 261-270)

Paragraph 5:

        e. Conclusion, recommendation and future direction (p.15, line: 274-279).

**Other changes to the manuscript:**

- The last sentence of the third paragraph within the Discussion section ‘*Results on the feasibility and trainability of our prehabilitation program will be submitted soon.*’ is changed into ‘*Results on the feasibility and trainability of our prehabilitation program are submitted.*’ (p.14, line: 260).

________________________________________

7. PLOS authors have the option to publish the peer review history of their article (what does this mean?). If published, this will include your full peer review and any attached files.

If you choose “no”, your identity will remain anonymous but your review may still be made public.

Do you want your identity to be public for this peer review? For information about this choice, including consent withdrawal, please see our Privacy Policy.

Reviewer #1: Yes: Salah Altarabsheh

Reviewer #3: Yes: Walid Kamal Abdelbasset
